# Left-right asymmetry in oxidative stress sensing neurons in *C. elegans*

**DOI:** 10.17912/micropub.biology.000652

**Published:** 2022-10-19

**Authors:** Sophie Quintin, Gilles Charvin

**Affiliations:** 1 IGBMC, Development and Stem Cells Department; 2 CNRS UMR7104, INSERM U964, Université de Strasbourg, 67404 France

## Abstract

Perception of oxidative stress in nematodes involves specific neurons expressing antioxidant enzymes. Here, we carefully characterized GFP knock-in lines for *C. elegans* peroxiredoxin PRDX-2 and thioredoxin TRX-1, and uncovered that left and right I2, PHA and ASJ neurons reproducibly express an asymmetric level of each enzyme. We observed that high-expressing neurons are in most cases associated with a particular side, indicating a directional rather than stochastic type of asymmetry. We propose that the biological relevance of this left-right asymmetry is to fine-tune H _2_ O _2_ or light sensing, which remains to be investigated.

**Figure 1. A left-right asymmetric level of antioxidant enzymes is observed in H 2 O 2 and light sensing neurons:**
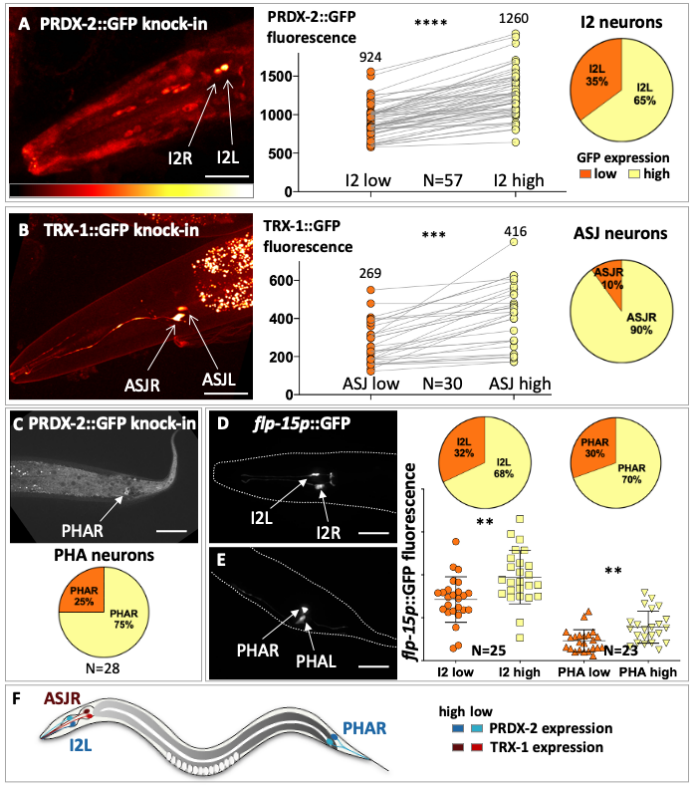
(A)- Spinning-disc confocal projection of the head of a representative PRDX-2::GFP knock-in animal. Bottom, look up table used to illustrate left-right neurons intensity differences (‘glow’ in Fiji, indicating a fluorescence intensity increase from left to right). In this and subsequent panels, animals are shown with anterior to the left and dorsal side up. I2L and I2R (arrows) exhibit PRDX-2::GFP asymmetric expression levels, as shown by the fluorescence intensity quantification (right panel). Pie chart, proportion of I2L neurons expressing low and high PRDX-2::GFP levels (B)- Spinning-disc confocal projection of the head of a representative TRX-1::GFP knock-in animal, shown in false colors as in A. ASJL and ASJR show asymmetric TRX-1::GFP expression, as indicated by the quantification (right panel). Pie chart illustrating that the vast majority of ASJR neurons display a higher TRX-1::GFP expression (in yellow). (C) Spinning-disc confocal projection showing PRDX-2::GFP expression in PHA/PHB tail neurons. Pie chart, proportion of PHAR neurons expressing low and high GFP. (D-E) Spinning-disc confocal projection of the head (D) and tail (E) regions of an animal expressing the *flp-15* ::GFP transcriptional reporter. Dashed line, outline of the animal, inferred from corresponding DIC image. Right panel, dot plot showing I2 and PHA unequal *flp-15p* ::GFP fluorescence levels. Top, fraction of I2L and PHAR neurons expressing low (orange) and high (yellow) GFP levels. (F) Sketch of a nematode summarizing the left-right asymmetric antioxidant enzyme levels in I2, ASJ and PHA neurons. The neuron of each pair with higher expression level of PRDX-2 or TRX-1 has been darkened for better contrast with the contralateral neuron. Means are represented and error bars indicate standard deviation in all graphs. N, number of animals quantified. p-values were calculated with an unpaired two-tailed Student test, Welch-corrected (A and B), or with a Mann Whitney test for PHA neurons (E) **p<0.01; ***p<0.001; ****p<0.0001. Scale bar, 100µm.

## Description

In multicellular organisms, perception of a broad range of H _2_ O _2_ concentrations is necessary to avoid oxidative stress in the environment. Recently, nematodes have been reported to accurately locate a safe niche with an appropriate H _2_ O _2_ level, by sensing H _2_ O _2_ through ASJ neurons, which mediate avoidance (Schiffer et al., 2021). ASJ neurons belong to the amphid sensillae and were previously identified as photosensory neurons (Liu et al., 2010; Ward et al., 2008). Nematodes possess additional pairs of H _2_ O _2_ and light-sensing neurons, such as I2 and PHA tail neurons, in which the H _2_ O _2_ response requires the activity of a neuronal antioxidant enzyme, the PRDX-2 peroxiredoxin (Bhatla and Horvitz, 2015; Quintin et al., 2022). In ASJ neurons, the thioredoxin TRX-1 is expressed (Miranda-Vizuete et al., 2006), and is essential for shaping the sensory response to nitric oxide (NO), which betrays the presence of pathogenic bacteria (Hao et al., 2018). Therefore, ASJ neurons could rely on TRX-1 for H _2_ O _2_ and NO sensing, while the I2 and PHA neurons response to H _2_ O _2_ depends on PRDX-2. Beyond their well-known peroxidase function, PRDX-2 and TRX-1 have also been shown to influence cellular signaling, and were proposed to act as H _2_ O _2_ sensors (Ledgerwood et al., 2017; Rhee and Woo, 2011; Veal et al., 2007). Interestingly, micromolar doses of H _2_ O _2_ promote neuronal sensory function (Li et al., 2016), while millimolar concentrations are deleterious for neurons (Gourgou and Chronis, 2016). Consequently, it is critical for nematodes to detect a broad range of H _2_ O _2_ concentrations to avoid noxious environments. Whether neuronal antioxidant enzyme levels play a role in this perception has not been investigated, but this question deserves attention in the future. Here, we sought to determine antioxidant levels in the H _2_ O _2_ or light-sensing I2, ASJ and PHA neurons.

To accurately monitor antioxidant enzyme levels in neurons, we generated C-terminal GFP knock-in versions of PRDX-2 and TRX-1 by supplying engineered templates for repair of CRISPR/Cas9-mediated cleavage (Dickinson et al., 2013; Quintin et al., 2022). Strikingly, we observed that I2 neurons reproducibly express an asymmetric mean intensity of PRDX-2::GFP expression in left and right neurons (Fig. 1A), in synchronized L4 and young adults. In 65% of animals (N=57), the high-expressing PRDX-2 neuron corresponded to I2L, while it was I2R in the remaining 35%. Similarly, we noticed an unequal level of TRX-1::GFP in ASJL and ASJR (Fig. 1B), and ASJR was associated with the highest TRX-1 intensity in 90% of animals (N=30), indicating a directional asymmetry. We next asked whether the nociceptive PHA neurons, which respond to light and H _2_ O _2_ (Quintin et al., 2022), also display such an asymmetry. In 75% of the PRDX-2::GFP knock-in line, only a single PHA neuron expressing PRDX-2::GFP was visible, the contralateral being undetectable, revealing an obviously asymmetric expression level but rendering quantification impossible (Fig. 1C). To examine this PHAL/R asymmetry with an independent marker, we quantified the neuropeptide *flp-15p* ::GFP transcriptional reporter specifically expressed in PHA and I2 neurons (Kim and Li, 2004). This analysis also revealed a significantly different left/right GFP level in PHA neurons, akin to that observed in I2 neurons (Fig. 1DE). Interestingly, the fraction of I2L and PHAR expressing the highest GFP signal was very similar (68% and 70%, respectively). We conclude that left-right asymmetric peroxiredoxin and neuropeptide levels are present in I2 and PHA neurons, while ASJ neurons show an asymmetric thioredoxin level. This asymmetry seems to be associated with a biased laterality as the proportion of high-expressing neurons is associated with a particular side; I2L, ASJR and PHAR displaying a higher expression in most cases (Fig. 1F). Interestingly, Goldsmith et al., (2010) have reported that the direction of ASER/ASEL size asymmetry is also variable, contrasting with the invariant left/right asymmetric expression of their chemoreceptors. Here, we favor the hypothesis that I2, ASJ and PHA neurons share the same type of directional asymmetry as ASE neurons.

In conclusion, we report a novel case of neuronal asymmetry in *C. elegans* , which is characterized by asymmetric expression levels of PRDX-2 or TRX-1 antioxidants in H _2_ O _2_ and light-sensing neurons (I2, ASJ and PHA, Fig 1). Left-right neuronal asymmetry has been reported to broaden the chemosensory repertoire of the nematode, notably in ASEL-R and AWC ^ON/OFF^ neurons, which exhibit distinct types of asymmetry (stereotyped versus stochastic; reviewed in (Hobert, 2014; Hsieh et al., 2014)). Beyond the well characterized ASE and AWC cases, the field of neuronal asymmetry is expanding. An asymmetric expression of the bHLH transcription factor HLH-16 has been reported in four pairs of interneuron and motoneurons of a navigation circuit, suggesting other cases of functional lateralization (Bertrand et al., 2011). More recently, five pairs of neurons of the ABa lineage were reported to be asymmetric in the expression levels of a C32C4.16::T2A::GFP reporter (Charest et al., 2020), revealing an unexpected degree of cellular diversification. Importantly, functional analyses have uncovered asymmetric contributions of left and right PLM neurons to the tail touch response, and pointed to their synergistic role in the response to weaker stimuli (Davis et al., 2021). Therefore, we suggest that unequal levels of antioxidant enzymes in left and right neurons could similarly result in differential functional properties. This hypothesis is reinforced by our observation that PHAL and PHAR show a small but significant difference in response to 10µM H _2_ O _2_ that is not seen at a higher dose (Quintin et al., 2022). Upon integration, they might allow nematodes to sense a broader range of environmental oxidative stressors, but this question needs to be further investigated.

## Methods

**Strains and genetics**

*C. elegans* strains (listed below) were grown on NGM plates as described (Brenner, 1974).

**CRISPR-Cas9 genome editing**

The PRDX-2::GFP strain was previously generated (Quintin et al., 2022) by CRISPR/Cas9-mediated ‘knock-in’ editing as described (Dickinson et al., (Bertrand et al., 2011)2013). For TRX-1 knock-in, we similarly used a long-arm based strategy with a guide RNA targeting a sequence located a few bp away from the stop codon (Dickinson et al., 2013). Phusion DNA polymerase was used to amplify by PCR 5’ and 3’ *trx-1* homology arms (HAs) from N2 genomic DNA, using primers containing SapI restriction sites and silent mutations to prevent Cas9 re-cleavage (listed in Reagents). A combined small guide-RNA/repair template plasmid was generated, after assembly of purified 5’ and 3’HAs and sgRNA oligonucleotides into the destination vector (pMLS256), together with the flexible linker (from pMLS287), GFP and the *unc-119* rescuing element (from pMLS252), in a single SapI restriction-ligation reaction, as described (Schwartz and Jorgensen, 2016). After transformation in DH5α cells, the pSQ7 plasmid was verified by restriction digest analysis and sequencing. All plasmids used for injection were purified using a DNA Miniprep Kit (PureLink, Invitrogen) or a DNA midiprep kit (Macherey Nagel). A plasmid mix containing combined sgRNA/repair template plasmid (pSQ7, 50 ng/μl), Cas9-encoding pSJ858 (25ng/μl) and co-injection markers (pCFJ90 at 2.5ng/μl; pCFJ104 at 5ng/μl, and pGH8 at 5ng/μl) was injected in the germline of *unc-119(ed3)* animals. Plates containing 2/3 injected F0 animals were starved, chunked on fresh plates, for candidates screening (attested by the presence of wild-type non fluorescent animals). Knock-in events were validated by PCR on homozygous lysed worms (QuantaBio AccuStart II GelTrack PCR SuperMix), using primers annealing in the inserted sequence and in an adjacent genomic region not included in the repair template.

**Spinning-disk confocal microscopy acquisitions and fluorescence intensity measurements**

Synchronized L4/young adults animals were anesthetized in M9 containing 1mM levamisole and mounted between slide and coverslip on 3% agarose pads. Spinning-disk confocal imaging was performed on an inverted DMI8 Leica microscope equipped with a Yokogawa CSUW1 head and an Orca Flash 4.0 camera, piloted by the Metamorph software. Objectives used were oil-immersion 40X (HC PL APO, NA 1.3) or 63X (HCX PL APO Lambda blue, NA 1.4). The temperature of the microscopy room was maintained at 20 ˚C for all experiments. Z-stacks of head region were acquired with a constant exposure time and a constant laser power in all experiments. Maximum intensity projections were used to generate the images shown. Fluorescence intensity measurements of I2, ASJ and PHA neurons were performed using the Fiji software, by drawing a region of interest (ROI) around neuronal cell body and average pixel intensity was quantified.

**Statistical analyses**

P-values were calculated with an unpaired two-tailed Student test; the Welch correction was applied when the two samples had unequal variances. A Mann Whitney test was made in Fig1E for PHA neurons as values did not pass normality tests. Error bars depict standard deviation in all graphs. Statistical analyses were conducted with the GraphPad Prism9 software. The data presented here come from at least three independent experiments. For p values, not significant p>0.05; *p<0.05; **p<0.01; ***p<0.001; ****p<0.0001.

## Reagents

**Strains**

**SXB15** *prdx-2* ( *gch01* [PRDX-2::GFP, *unc-119* (+)] II outcrossed 5 times (Quintin et al., 2022)

**SXB34** *trx-1* ( *gch09* [TRX-1::GFP, *unc-119* (+)] II; *unc-119(ed3)* III (this study)

**HT1593** 7 times outcrossed *unc-119(ed3)* III CGC

**Primers for TRX-1::GFP repair template (pSQ7 plasmid)**

All primers sequences are listed in the 5’-3’orientation.

1- Introduction of *trx-1* sgRNA (into pMLS256), targeted 10 bp after the TRX-1 stop codon:

**Top oligo** : 5’- **TTG** ATGATTGGTTTAAAAGCAGA

**Bottom oligo** : 5’- **AAC** tctgcttttaaaccaatcat

2- PCR amplification of 5’ and 3’ homology arms (HAs) of *trx-1* (including a **SapI restriction** site and template-specific **overhangs** ):

— 5’ HA PCR product (560bp), ending right before the TRX-1 stop codon:

**Forward:** 5’ **-** tct **GCTCTTC** t **TGG** TGCGGACCATGCAAAGCAATTGCA

**Reverse** : 5’- atg **GCTCTTC** a **CGC** TTGAGCAGATACGTGCTCCAACACT

— 3’ HA PCR product (439bp), starting at the TRX-1 stop codon:

**Forward:** 5’- acg **GCTCTTC** tG **GTT** GAtcttcga **TC** atctgct **A** ttaaaccaatcatctgctcattgagctc **AA** ccgct (including **mutations** in the PAM, sgRNA sequences and in a SapI site present in the sequence)

**Reverse** : 5’-ttc **GCTCTTC** a **TAC** aaagccatcaaagaactgggacttctga
